# Pollinator dependence but no pollen limitation for eight plants occurring north of the Arctic Circle

**DOI:** 10.1002/ece3.6884

**Published:** 2020-11-28

**Authors:** Viviane Koch, Leana Zoller, Joanne M. Bennett, Tiffany M. Knight

**Affiliations:** ^1^ Institute of Ecology University of Bremen Bremen Germany; ^2^ Institute of Biology Martin Luther University Halle‐Wittenberg Halle (Saale) Germany; ^3^ German Centre for Integrative Biodiversity Research (iDiv) Halle‐Jena‐Leipzig Leipzig Germany; ^4^ Centre for Applied Water Science, Institute for Applied Ecology, Faculty of Science and Technology University of Canberra Canberra ACT Australia; ^5^ Department of Community Ecology Helmholtz Centre for Environmental Research‐UFZ Halle (Saale) Germany

**Keywords:** arctic, high latitude, plant–pollinator interactions, pollen limitation, pollination, pollinator dependence

## Abstract

Effective interactions between plants and pollinators are essential for the reproduction of plant species. Pollinator exclusion experiments and pollen supplementation experiments quantify the degree to which plants depend on animal pollinators and the degree to which plant reproduction is pollen limited. Pollen supplementation experiments have been conducted across the globe, but are rare in high latitude regions. To fill this knowledge gap, we experimentally investigated the dependence on animal pollinators and magnitude of pollen limitation in eight plant species north of the Arctic Circle in Lapland, Finland. Our findings show that all plant species were pollinator dependent, but not pollen limited. We discuss several mechanisms that might buffer our focal plants from pollen limitation, including plant and pollinator generalization, and attractive plant traits. Our results demonstrate that many plant species north of the Arctic Circle are currently receiving adequate pollinator service and provide a baseline for future comparisons of pollinator dependence and pollen limitation in the Arctic across space and time.

## Graphical abstract

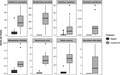

## 1 INTRODUCTION

Globally, ~87.5% of the angiosperms rely on pollination by animals, at least to some extent, to ensure their reproduction (Ollerton et al., 2011). Thus, plant reproduction might be limited if plants receive inadequate quantity or quality of pollen from their animal pollinators (Bennett et al., 2018; Knight et al., 2005). Pollen supplementation experiments quantify the magnitude of pollen limitation. In these experiments, a saturating amount of outcross pollen is added to the stigma of flowers and the reproductive success of these pollen supplemented flowers is compared to that of naturally pollinated flowers (Bennett et al., 2018; Knight et al., 2005). Results of a global meta‐analysis of 2,969 experiments found high variation in pollen limitation across plant species and across locations (Bennett et al., 2018). For example, invasive plants are less pollen limited than native plants (Burns et al., 2019) and plants in urban sites are more pollen limited than those occurring in more natural sites (Bennett et al., 2020).

Our global knowledge of the extent and magnitude of pollen limitation is currently limited by unequal sampling across the world's biomes. For example, only 27 of the 2,969 pollen supplementation experiments synthesized in a recent meta‐analysis were conducted in a polar biome under the Koeppen–Geiger biome classification (Bennett et al., 2018), with only one experiment conducted above the Arctic Circle (in western Greenland; Urbanowicz et al., 2018). Thus, high latitude locations represent a knowledge gap in the field of pollination ecology.

Higher latitudes are experiencing an accelerated rate of increasing temperatures compared with lower latitude regions (Post et al., 2009). This rapid climate change can increase or decrease pollen limitation for pollinator‐dependent plants (see Hegland et al., 2009; Scaven & Rafferty, 2013), depending on the plant´s access and response capacity to changing pollinator services (Burkle & Alarcon, 2011). For example, climate change can (a) create spatial mismatches between plants and their pollinators (Hegland et al., 2009), if one of the mutualistic interaction partners moves or goes extinct when climate exceeds its physiological tolerances (Kerr et al., 2015); (b) create temporal mismatches between previously interacting species due to phenological changes, and allow for new interactions between species that did not previously interact (Burkle et al., 2013; Hegland et al., 2009); and (c) result in movement of plants and/or pollinators toward the poles, which could increase the richness and/or abundance of pollinator species at higher latitudes (Franzén & Öckinger, 2012; Parmesan, 2006).

Plant mating systems such as autogamy (self‐fertilization) are known to buffer plant species from pollen limitation (Knight et al., 2005). Autogamous plants that are able to fully reproduce even in the absence of visits from animal pollinators are less prone to pollen limitation than plants with outcrossing mating systems (Bennett et al., 2020; Knight et al., 2005). The distributional pattern of autogamous plant mating systems across the globe is likely in response to low pollinator abundances or effectiveness of pollinators at some locations (Grossenbacher et al., 2015; Moeller et al., 2017; Ollerton et al., 2011). For example, plant species show patterns of less pollinator dependence (i.e., more autogamy) moving from tropical to the temperate latitudes (Ollerton et al., 2011). Autogamy occurs more frequently at the edge of geographic ranges of plant species (including higher latitudes) (Grossenbacher et al., 2015; Hargreaves & Eckert, 2014). However, there is high variation among plants in their pollinator dependence, even at high latitudes (Moeller et al., 2017), and there are still many pollinator‐dependent plants in high latitude locations (Kevan, 1972). Thus, it is important to simultaneously quantify both animal pollinator dependence and pollen limitation in high latitude sites.

Other plant traits, such as floral phenotype and floral rewards, are expected to buffer plants from pollen limitation. Plants that are phenotypically and ecologically generalized in their pollination (e.g., actinomorphic flowers that are visited by many species of pollinators) tend to show lower pollen limitation than those that are more specialized (e.g., Wolowski et al., 2014), likely because they are less sensitive to variation in pollinator communities across space and time (Bartomeus et al., 2011; Burkle & Alarcon, 2011; Burns et al., 2019). Moreover, rewarding plant species (i.e., nectariferous) also tend to show lower levels of pollen limitation than unrewarding species (i.e., nectarless; Larson & Barrett, 2000). Thus, in high latitude sites, plant species that are phenotypically and ecologically generalized and/or offer rewards might avoid pollen limitation.

Our study examines the animal pollinator dependence and the magnitude of pollen limitation for eight plant species occurring north of the Arctic Circle in Lapland, Finland. We conduct a pollinator exclusion treatment and a pollen supplementation treatment to quantify the degree to which each plant species relies on animal pollinators for reproduction and to quantify the magnitude of pollen limitation.

## 2 MATERIALS AND METHODS

### 2.1 Study sites and plant species

The study was conducted in a ~ 7 km radius around Kittilä (67°40′0.01″N, 24°54′0″E) in Lapland, Finland. The study region is located north of the Arctic Circle in a boreal biome. Sites were in a range of sun‐exposed habitats, including meadows, birch‐dominated forests, sandy riparian habitats, and bogs. In general, the sites are near‐natural, as this region has low human population density, few invasive plant species, and little agricultural land use. All focal plant species were studied at one site, except for *Vaccinium vitis‐idaea* and *Silene suecica* which were each located at two sites in close proximity to each other (Table 1) as there were too few individuals at any one site.

**TABLE 1:** Focal plant species, their general characteristics, and the location of our study sites. Information on flowering and seed ripening period and mating system comes from the BIOLFLOR database (Klotz et al., 2002)

Species	Family	Flowering period	Seeds ripen from	Mating system	Pollination syndrome	References	Main observed visitors	Coordinates of study sites
*Anthriscus sylvestris*	Apiaceae	May–June	July	Sexual, amphimictic (self‐compatible)	Diptera	Van Mierlo & van Groenedael (1991), Darbyshire et al. (1999)	Flies, syrphids, sawflies	N 67° 39.998′ E 024° 53.109′
*Dactylorhiza maculata*	Orchidaceae	June–August	July	Sexual, amphimictic (self‐compatible)	Bumblebees, syrphids	Koivisto et al. (2002), Vallius (2000, 2001)	Flies, syrphids	N 67° 39.605′ E 024° 46.030′
*Dianthus superbus*	Caryophyllaceae	June–September	July	Sexual, amphimictic (self‐compatible)	Moths (sphingids, noctuids), lepidoptera	Jürgens et al. (2002)	Flies, bumblebees, moths	N 67° 38.913′ E 024° 55.136′
*Geranium sylvaticum*	Geraniaceae	June–July	July	Sexual, amphimictic (self‐compatible)	Syrphids, bumblebees, diptera	Asikainen and Mutikainen (2005)	Flies, syrphids, bumblebees	N 67° 41.441′ E 024° 54.568′
*Menyanthes trifoliata*	Menyanthaceae	May–July	June	Sexual, amphimictic (self‐incompatible)	Lepidoptera, diptera, hymenoptera	Van Rossum et al. (2015), Thompson et al. (1998)	Flies, syrphids, bumblebees	N 67° 36.171′ E 024° 56.698′
*Ranunculus acris*	Ranunculaceae	May–July	July	Mostly sexual, rarely apomictic (self‐incompatible)	Syrphids, bumblebees, flies	Totland (1994), Jakobsson et al. (2009)	Flies, syrphids	N 67° 41.434′ E 024° 54.540′
*Silene suecica*	Caryophyllaceae	June–August	July	Mostly sexual	Lepidoptera, bees, flies, syrphids	Jürgens et al. (2002)	Flies, syrphids, bumblebees	N 67° 41.442′ E 024° 54.671′; N 67° 41.442′ E 024° 54.644′
*Vaccinium vitis‐idaea*	Ericaceae	June August	August	Sexual, amphimictic (self‐compatible)	Bumblebees	Jaquemart and Thompson (1996), Nuortila et al. (2002)	Bumblebees	N 67° 41.442′ E 024° 54.671′; N 67° 41.446′ E 024° 54.685′

Note

Information on pollination syndrome comes from the references in the table. Main observed visitors are personal observations made in the field by VK and LZ.

We selected 12 plant species to investigate pollinator dependence (i.e., dependence on animal pollinators) and pollen limitation; however, we concluded the study with eight species due to loss of replicates from human disturbances (i.e., mowing) and herbivory/ parasitism. We chose plant species that were available for study during our field season (i.e., flowered in the early summer and would mature seeds and fruits in late summer) and that represented a range of different plant families and pollination syndromes (Table 1). Three of eight plant species, *Ranunculus acris*, *Vaccinium vitis‐idaea,* and *Geranium sylvaticum*, are known to be pollinator dependent in other regions (Lundgren et al., 2013; Nuortila et al., 2002; Totland, 1994). The other five plant species, *Anthriscus sylvestris*, *Dactylorhiza maculata, Dianthus superbus*, *Silene suecica,* and *Menyanthes trifoliata,* have not been identified as pollinator dependent or autofertile. However, these species are known to be insect pollinated and *Menyanthes trifoliata* is heterostylous (Darbyshire et al., 1999; Kostrakiewicz‐Gieralt, 2013; Mondoni et al., 2018; Olesen, 1987; Thompson et al., 1998; Vallius, 2000). This study provides the first experimental data on pollinator dependency for these five species.

### 2.2 Pollinator exclusion and pollen supplementation experiments

In the early summer of 2019 (except *Menyanthes trifoliata*, which was studied in 2018), we randomly selected at least 30 flowering plants of each plant species (except *Dactylorhiza maculata* because we only found 15 flowering individuals) with similar number of buds/ flowers and similar height of individuals. We randomly assigned each plant to one of three pollination treatments: bagged (B), natural (N), and supplement (S), resulting in 1/3 of the total individuals of each plant species in each treatment. In the bagged treatment, all the flowers, umbels, or inflorescence of an individual were bagged before the flowers opened to exclude all animal pollinators from visiting. Bags were made of light‐weight material (“Organza”) with a mesh size of ~ 0.5mm, which excludes animal pollinators but still allows wind, rain, and sun to enter. Pollination by wind can therefore not be ruled out. In the supplement treatment, outcrossed pollen was added to each flower, umbel, or inflorescence of an individual by using a brush or by rubbing the anthers from a single flower/umbel of a donor individual onto the stigma of the flowers of the focal plant species. Flowers in the supplement treatment were also open to natural pollination. The outcrossed pollen was collected from a flower at least 10 meters away from the focal plant to minimize the risk of inbreeding (Charpentier et al., 2000). The treatments were applied to all flowers of an individual to avoid resource reallocation, which can inflate estimates of pollen limitation (Knight et al., 2006). Plants with consecutively opening flowers were revisited almost daily (depending on weather condition) until all flowers of the inflorescence had flowered and each flower had received supplemental pollen. Plants in the natural treatment were open to natural pollination and not manipulated in any way.

Mature (and semimature) fruits were collected from all plants, and the number of viable seeds per fruit was counted. We considered following metrics of plant reproductive success: (a) fruit set (mean fruits per flower), (b) mean seeds per fruit, and (c) mean seeds per flower. As seeds per flower are the product of fruit set and mean seeds per fruit, this is the most comprehensive response variable. While our treatments were applied to entire plants, and effort was made to distribute treatments across plants of similar size, we do not report seeds per plant due to differences in flower production across individual plants. We provide data from all three metrics in the supplementary data file.

### 2.3 Statistical analyses

We tested for pollinator dependence and pollen limitation for each of our species and metrics using two statistical approaches: (1) one‐way ANOVA with Tukey's multiple comparisons and (2) Kruskal–Wallis multiple comparison test with adjusted *p*‐values using the Benjamini–Hochberg method. ANOVA is the most common analysis technique used to assess pollen limitation (Bennett et al., 2018). However, our data are not normally distributed. Although ANOVA is fairly robust to the violation of this assumption (Lix et al., 1996), the nonparametric Kruskal–Wallis test which does not assume normality was more appropriate for this study. The Kruskal–Wallis test was performed for 8 species and 3 pairwise treatment comparisons for each metric, and therefore, it was necessary to adjust *p*‐values for multiple comparisons. Due to its common use, we also provided Tukey's results in text using untransformed data. Transformation did improve normality to some degree, but we found no quantifiable difference between models using untransformed and transformed data. Both statistical approaches provided consistent results. All analyses were performed in the statistical program R 3.6.4 (R Core Development Team, 2020) using the standard base functions.

## 3 RESULTS

### 3.1 Pollinator dependency

**FIGURE 1:**
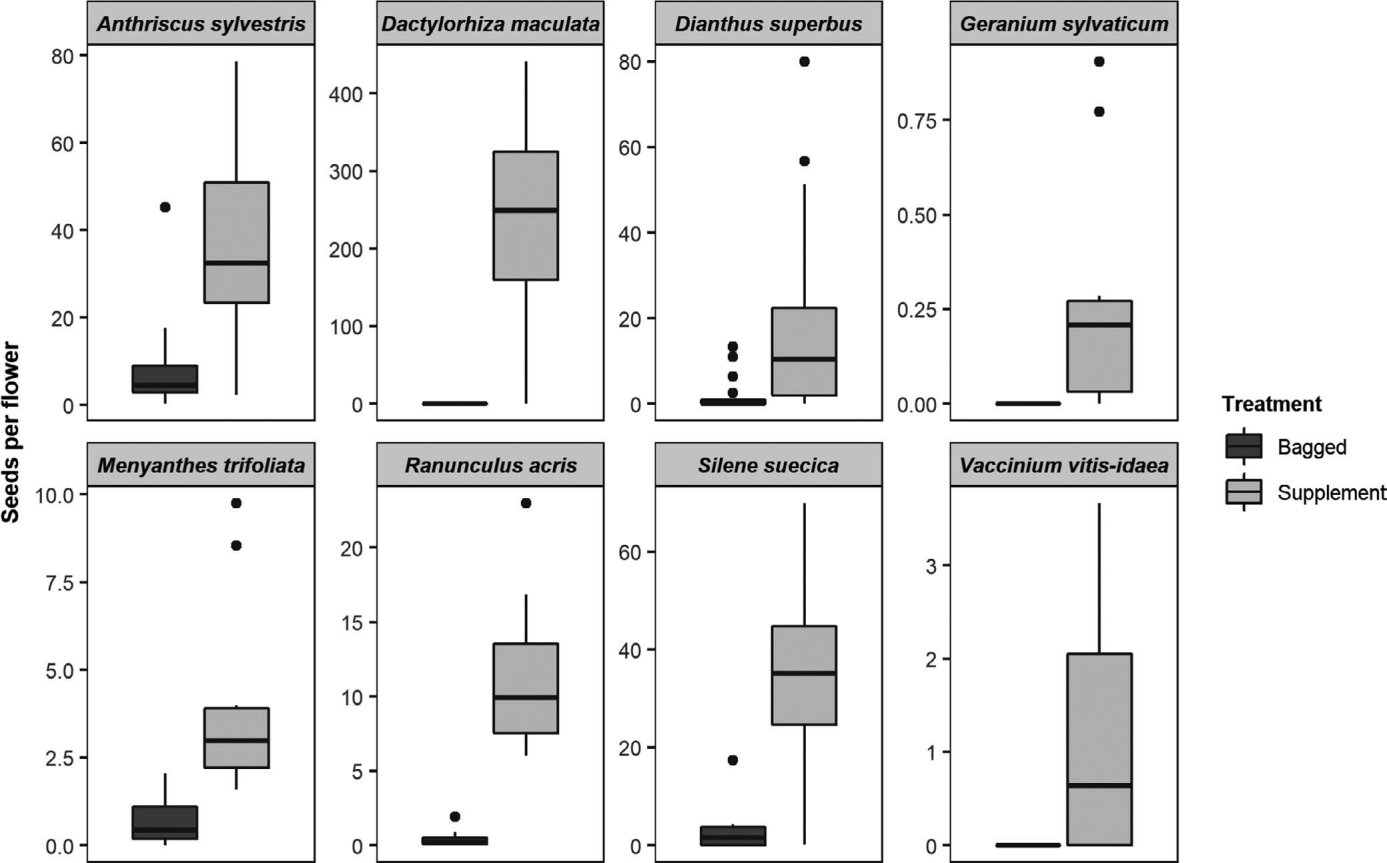
All plant species are pollinator dependent. Boxplots show the median, 25%‐75% interquartile range and the “minimum” and “maximum” (lines) for seeds per flower in Bagged (B) versus Supplement (S) treatments for each of the eight focal plants species. Black points represent outliers

For the most comprehensive metric, seeds per flower, there was higher seed production in the supplement compared with the bagged treatment for all species, indicating significant dependence on animal pollinators (Tables 2 and 3, Figure 1). This difference between supplement and bagged treatments was also seen for most species for the other two metrics, fruit set and seeds per fruit (Table S1). *Vaccinium vitis‐idaea* and *Dactylorhiza maculata* plants produced no fruits in the bagged treatment (Table 3), and thus, it was not possible to calculate the seeds per fruit for these species (Table S1). *Anthriscus sylvestris* produced fewer fruits when pollinators were excluded but displayed no significant difference between treatments in seeds per fruit, as each Schizocarp fruit splits into two Mericarps containing one viable seed each (Table S1). *Ranunculus acris* showed no difference between the treatments in fruit set but did produce fewer seeds per fruit when excluded from pollinators (Table S1).

**TABLE 2:** Results from a Kruskal–Wallis multiple comparison test with adjusted *p*‐values using the Benjamini–Hochberg method with seeds per flower as the response variable and treatment as the predictor

Species	N‐B	S‐B	S‐N
*Anthriscus sylvestris*	3.96****	2.62**	−1.41
*Dactylorhiza maculata*	1.79	2.51**	0.65
*Dianthus superbus*	4.70****	3.45****	−1.31
*Geranium sylvaticum*	3.88****	2.70***	−1.05
*Menyanthes trifoliata*	2.97**	3.25***	0.95
*Ranunculus acris*	4.85****	3.53****	−1.31
*Silene suecica*	3.69****	3.62****	−0.08
*Vaccinium vitis‐idaea*	1.85*	3.00***	1.10

Note

Treatments are as follows: supplement (S), natural control (N), and bagged (B). The *z* value is shown for each comparison. Significant differences between treatment pairs based on adjusted *p*‐values are highlighted in bold and the level of significance annotated as *<.1, **<.05. ***<.01,****<.001. All plant species were significantly pollinator dependent (S‐B), and no species was pollen limited (S‐N).

**TABLE 3:** The mean seeds per flower, one standard error (SE), and sample size (n) for the three treatments: supplement (S), natural control (N), and bagged (B) and the mean difference from a Tukey test

Species	S mean ± *SE* (*n*)	*N* mean ± *SE* (*n*)	B mean ± *SE* (*n*)	N‐B	S‐B	S‐N
*Anthriscus sylvestris*	37.4 ± 6.52 (12)	52.9 ± 6.78 (12)	9.69 ± 4.28 (10)	43.2****	27.7*	−15.5
*Dactylorhiza maculata*	235 ± 91.67 (4)	107 ± 48.9 (4)	0 ± 0 (6)	107	235	128
*Dianthus superbus*	17.7 ± 5.17 (19)	26.6 ± 6.09 (18)	1.93 ± 6.09 (18)	24.6***	15.8*	−8.87
*Geranium sylvaticum*	0.273 ± 0.10 (10)	0.541 ± 0.15 (11)	0 ± 0 (12)	0.541***	0.273	−0.268
*Menyanthes trifoliata*	3.98 ± 0.92 (10)	3.70 ± 0.80 (9)	0.73 ± 0.20 (11)	3.55****	3.77****	0.11
*Ranunculus acris*	11.2 ± 1.45 (12)	14.0 ± 0.77 (12)	0.414 ± 0.16 (12)	13.6****	10.8****	−2.81
*Silene suecica*	36.0 ± 5.91 (12)	34.2 ± 3.16 (12)	3.22 ± 1.67 (10)	31.0****	32.8****	1.78
*Vaccinium vitis‐idaea*	1.19 ± 0.47 (10)	0.654 ± 0.48 (10)	0 ± 0 (12)	0.654	1.19*	0.538

Note

Results highlighted in bold are significant based on the adjusted *p*‐values and annotated according to significance level, *<.1, **<.05,***<.01,****<.001. All plants are pollinator dependent showed by a significant difference between either N‐B, S‐B or producing zero fruits and seeds when bagged. No plants were pollen limited (S‐N).

### 3.2 Pollen limitation

**FIGURE 2:**
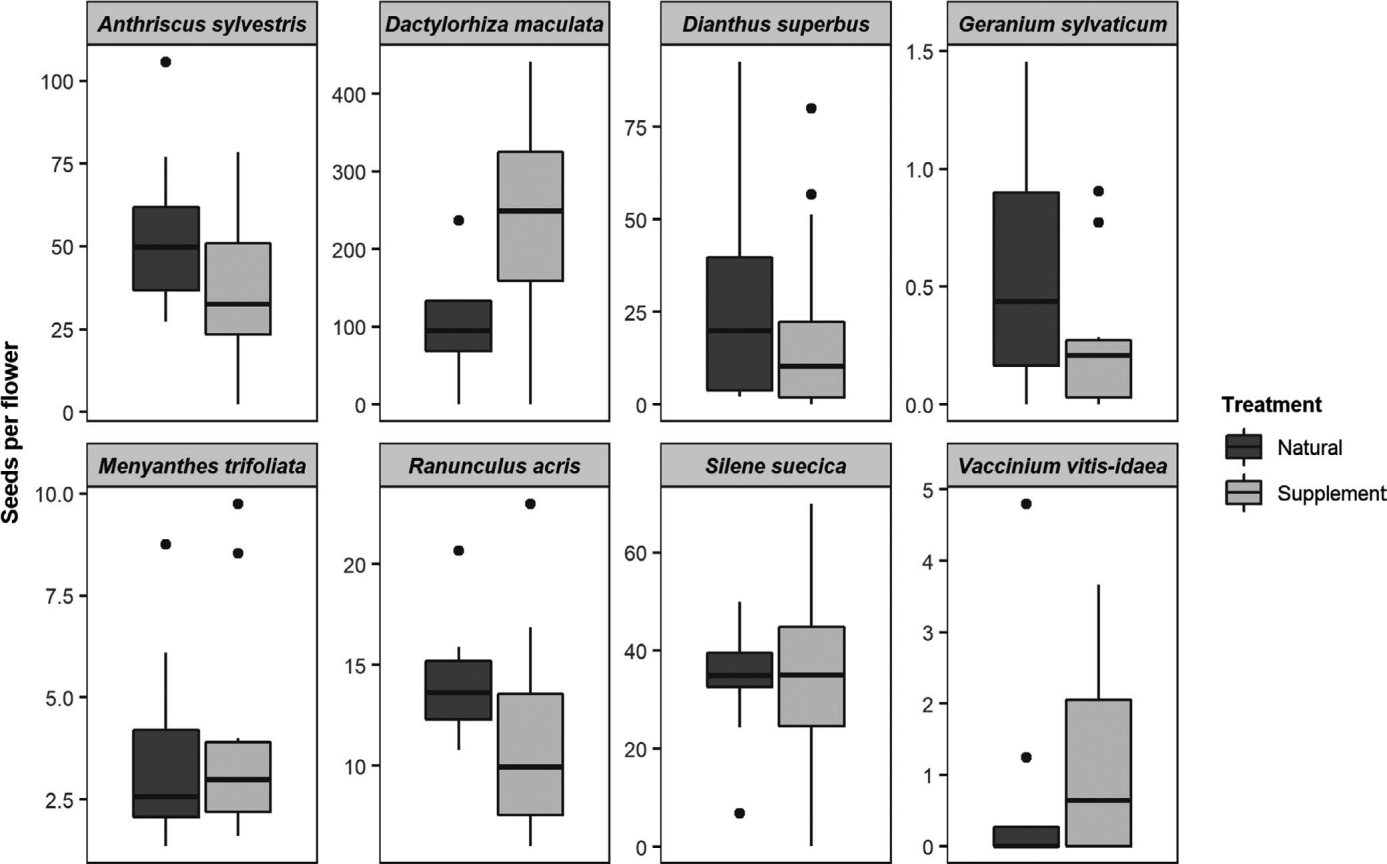
None of the plant species are pollen limited. Boxplots show the median, 25%–75% interquartile range and the “minimum” and “maximum” (lines) for seeds per flower in Natural (N) versus Supplement (S) treatments for each of the eight focal plants species. Black points represent outliers

None of the plant species showed significantly higher reproductive success in the supplement (S) compared with the natural treatment (N) in any reproductive metric, indicating that none of the plant species were pollen limited (Figure 2, Tables 2 and 3, and Table S1). *Dactylorhiza maculata* flowers had on average two times the fruit set in the supplement compared with the control treatments; however, low sample sizes prevented the detection of a significant effect (Table 3).

## 4 DISCUSSION

We found that all eight of our focal plant species occurring at this high latitude site depend on animal pollinators but are not pollen limited. Theory suggests that pollen limitation might intensify in locations with rapidly changing climate, due to spatial and temporal mismatches between plants and pollinators (Hegland et al., 2009). However, our results suggest that other mechanisms are counteracting these changes to maintain successful pollinator–plant interactions and reproduction at these latitudes (Knight et al., 2018).

Despite global reviews suggesting that autofertility may increase with latitude (Ollerton et al., 2011), we found all of our focal plant species to be pollinator dependent. However, it is important to note that Ollerton et al. (2011) were unable to include Arctic studies in their review due to a lack of available data. Another global review found latitude to be a weak predictor of plant mating system and suggested that plant phylogeny is a more important predictor of plant mating systems (Moeller et al., 2017). Three of our focal plant species, *Ranunculus acris*, *Vaccinium vitis‐idaea,* and *Geranium sylvaticum,* were previously identified as pollinator dependent in more southern locations (Lundgren et al., 2013; Nuortila et al., 2002; Totland, 1994). Our study provides the first experimental data on pollinator dependency for the other five plant species: *Anthriscus sylvestris*, *Silene suecica*, *Dianthus superbus*, *Menyanthes trifoliata,* and *Dactylorhiza maculata*.

We did not detect pollen limitation for any of our focal plant species, although our data provide a weak test in the case of *Dactylorhiza maculata*. This is surprising, as pollen limitation is common for pollinator‐dependent plants (Bennett et al., 2018; Burd, 1994; Knight et al., 2005). Further, some of our focal plant species showed pollen limitation in other regions of their range. For instance, *Geranium sylvaticum* showed pollen limitation, albeit low, in southern Finland (Asikainen & Mutikainen, 2005). *Vaccinium vitis‐idaea* showed a significant increase in fruit set after receiving supplemental pollination in Belgium (Jaquemart & Thompson, 1996). *Ranunculus acris* is a well‐studied plant and showed variable levels of pollen limitation across different regions and altitudes (Hegland & Totland, 2007; Jakobsson et al., 2009; Totland, 2001). One possible reason for this is that the cold temperature experiences in our high latitude region may constrain plant reproductive success more than pollen availability (Totland, 2001) by, for example, constraining photosynthetic activity.

To our knowledge, our study is the first to quantify pollen limitation for five of our focal species (*Menyanthes trifoliata, Anthriscus sylvestris, Silene suecica, Dianthus superbus,* and *Dactylorhiza maculata*). We had a small number of flowering individuals to work with for *Dactylorhiza maculata*, and while this species was not significantly pollen limited, our test was weak. Orchidaceae is one of the most pollen limited plant families in the world (Bennett et al., 2018; Knight et al., 2005; Larson & Barrett, 2000). Further, as a nectarless species with specialized floral traits (Vallius, 2000), *Dactylorhiza maculata* should be vulnerable to pollen limitation (Neiland & Wilcock, 1998; Wolowski et al., 2014).

There are a number of possible reasons for the absence of pollen limitation in our focal plant species. First, many of our focal species are phenotypically and ecologically generalized in their pollination, which should buffer them from pollen limitation in temporally changing high latitude locations. Further, some of our more specialized species are nectar rewarding (e.g., *Dianthus superbus,* Jürgens et al., 2002). Second, new interactions with migrating pollinators toward higher latitudes might buffer our focal plant species from pollen limitation. Climate is rapidly changing in our study region, Kittilä, with positive temperature anomalies in 18 of the past 20 years compared with baseline values (Finnish Meteorological Institute (FMI), 2020). Increasing temperatures, induced by climate change, are expected to result in range expansion and immigration of pollinators toward the poles (Kerr et al., 2015; Post et al., 2009).

Finally, it is possible that land use change might be more detrimental to pollination than climate change. Previous findings show that land use changes such as agricultural intensification, habitat fragmentation, and urbanization affect pollination service negatively, which might in turn cause pollen limitation in plants (Aguilar et al., 2006; Cunningham, 2000; Knight et al., 2018). A recent meta‐analysis shows that globally, plants in urban habitats are nearly twice as pollen limited as plants that occur in natural environments (Bennett et al., 2020). At our site, there have been little urbanization and agricultural activities, hence limited habitat fragmentation and pesticide use. This low anthropogenic activity in our research region might explain the almost complete absence of pollen limitation in our focal plants.

We found pollinator dependence but no pollen limitation in all of our focal plants. Our finding that our focal plants at this high latitude site do not show any pollen limitation is an optimistic result, but should be interpreted with caution. Although there are no indications of pollen limitation at the present day, this does not mean that climate change will not endanger the pollination and reproduction of plants in the future. Our results provide a baseline to examine how pollen limitation of our focal plant species might change across space in time with land use and climate change. There is a need to quantify the identity and abundance of visiting pollinators and pollination services to plants to understand the causes and consequences of pollen limitation or lack thereof. By providing the first experimental pollen limitation data for five of the plant species and one plant families (Menyanthaceae), our data contribute to the global knowledge on plant reproduction and plant–pollinator interactions.

## CONFLICT OF INTEREST

The authors declare that they have no conflict of interest.

## AUTHOR CONTRIBUTION

**Viviane Koch:** Data curation (equal); Formal analysis (supporting); Investigation (lead); Visualization (equal); Writing‐original draft (lead); Writing‐review & editing (equal). **Leana Zoller:** Conceptualization (equal); Data curation (equal); Formal analysis (supporting); Investigation (supporting); Visualization (equal); Writing‐original draft (supporting); Writing‐review & editing (equal). **Joanne M. Bennett:** Conceptualization (equal); Formal analysis (lead); Supervision (supporting); Visualization (supporting); Writing‐original draft (supporting); Writing‐review & editing (equal). **Tiffany M. Knight:** Conceptualization (equal); Formal analysis (supporting); Funding acquisition (lead); Project administration (lead); Resources (lead); Supervision (lead); Visualization (supporting); Writing‐original draft (supporting); Writing‐review & editing (equal).

## Supporting information

Table S1

## Data Availability

Data from the pollen exclusion experiments and the pollen supplementation experiments are available on Dryad: https://doi.org/10.5061/dryad.t76hdr7xk. The spreadsheet contains the total number of flowers, fruits, and viable seeds as well as the ratios of number of fruits per flower, number of seeds per fruit, and number of seeds per flower for the three treatments for eight plant species.
