# Supplementary material for: Pollinator dependence but no pollen limitation for eight plants occurring north of the Arctic Circle
Source: Ecol Evol. 2020 Nov 28;10(24):13664–72. doi: 10.1002/ece3.6884 (PMC7771129; doi:10.1002/ece3.6884)
Supplement: Supplementary file 1 — Table S1 [file ECE3-10-13664-s001.docx]

|  | Seeds per fruit | | | Fruits per flower | | |
| --- | --- | --- | --- | --- | --- | --- |
| Species | S mean ± SE (n) | N mean ± SE (n) | B mean ± SE (n) | S mean ± SE (n) | N mean ± SE (n) | B mean ± SE (n) |
| *Anthriscus sylvestris* | 0.5 ± 0  (12) | 0.5 ± 0  (12) | 0.5 ± 0  (10) | 74.8 ± 13.05  (12) | 106 ± 13.6  (12) | 19.4 ± 8.55  (10) |
| *Dactylorhiza maculata* | 1483 ± 141  (3) | 1173 ± 219  (3) | 0  (0) | 0.17 ± 0.05  (5) | 0.09 ± 0.03  (4) | 0 ± 0  (6) |
| *Dianthus superbus* | 19.7 ± 5.29  (19) | 28.2 ± 5.97  (18) | 2.47 ± 1.25  (18) | 0.91 ± 0.05  (19) | 0.88 ± 0.05  (18) | 0.71 ± 0.07  (18) |
| *Geranium sylvaticum* | 0.772 ± 0.22  (9) | 1.56 ± 0.38  (10) | 0 ± 0  (4) | 0.384 ± 0.08  (10) | 0.372 ± 0.09  (11) | 0.04 ± 0.02  (12) |
| *Menyanthes trifoliata* | 5.06 ± 0.92  (10) | 5.03 ± 0.71  (9) | 2.54 ±0.62  (11) | 0.76 ± 0.05  (10) | 0.684 ± 0.05  (9) | 0.269 ± 0.06  (11) |
| *Ranunculus acris* | 13.9 ± 1.67  (12) | 16.9 ± 1.33  (12) | 0.509 ± 0.19  (12) | 0.808 ± 0.04  (12) | 0.849 ± 0.04  (12) | 0.809 ± 0.04  (12) |
| *Silene suecica* | 41.0 ± 6.11  (12) | 37.4 ± 3.12  (12) | 5.99 ± 2.02  (9) | 0.887 ± 0.06  (12) | 0.901 ± 0.03  (12) | 0.375 ± 0.10  (10) |
| *Vaccinium vitis-idaea* | 5.54 ± 0.90  (6) | 5.25 ± 1.89  (4) | 0  (0) | 0.212 ± 0.08  (10) | 0.120 ± 0.08  (10) | 0 ± 0  (12) |

Table S1. The mean ± 1 SE and sample size (n) for seeds per fruit and fruits per flower for the three treatments: supplement (S), natural control (N), and bagged (B) in our eight focal species
